# Supplementary material for: Nested helicoids in biological microstructures
Source: Nat Commun. 2020 Jan 13;11:224. doi: 10.1038/s41467-019-13978-6 (PMC6957508; doi:10.1038/s41467-019-13978-6)
Supplement: Supplementary file 1 — Supplementary Information [file 41467_2019_13978_MOESM1_ESM.pdf]

Supplementary Information

Nested helicoids in biological microstructures

Greenfeld et al.

Supplementary Note 1. Geometric model mathematical description. This note provides the mathematical description of the geometric model. Refer to the *geometric model* section in the main text, including Figures 4, 5 and 6. Given the angular range of the twist and tilt angles, 2Θ and 2Φ, respectively, and assuming uniform angular steps between laminae (other assumptions are also possible), the angular increment between successive laminae is (Figure 1)

$$\delta_\theta = \frac{2\Theta}{n-1} \quad \text{and} \quad \delta_\phi = \frac{2\Phi}{n-1} \tag{1}$$

The sign of Θ determines the helical sense, positive for right-handed helicoid and negative for left-handed helicoid, whereas the sign of Φ is positive. Numbering the laminae from 1 at the bottom to n at the top, the twist and tilt angles of lamina k are given by

$$\begin{aligned} \theta_k &= -\Theta + (k-1)\delta_\theta \\ \phi_k &= -\Phi + (k-1)\delta_\phi \end{aligned} \tag{2}$$

Given the lamina thickness t, assumed to be uniform, the height of the BLU (Bouligand laminate unit) is $h = nt$ (excluding the contribution of tilt), and the z-position at the mid-thickness of lamina k is

$$z_k = kt - \frac{t}{2} - \frac{h}{2} = \frac{t}{2}(2k - n - 1) \tag{3}$$

at its intersection with the z-axis. As observed, the lamina consists of tightly packed unidirectional chitin fibers embedded in a proteinaceous matrix, and therefore t is equivalent to the fiber diameter and the matrix does not add to it but just fills the gaps between fibers. For any lamina k, located at z_k in the top half of the BLU, twisted θ_k and tilted ϕ_k, corresponds a lamina $n - k + 1$, located at $-z_k$, twisted $-\theta_k$ and tilted $-\phi_k$ (Figure 5a). Because of the tilt rotation, points on the lamina are either above or below z_k.

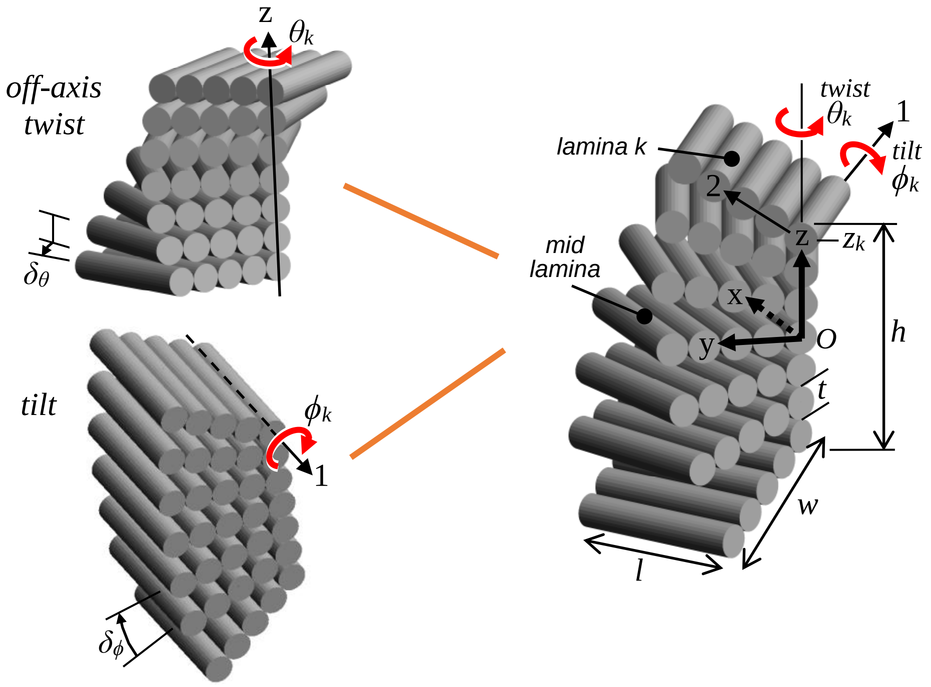

Figure 1. BLU (Bouligand laminate unit) geometrical model. *Isolated twist and tilt rotations (left) and resulting combined structure (right). Coordinate systems – layer/laminate* (x, y) *and lamina* $(1,2)$*; lamina k twist angle is* θ_k *with angular increment* δ_θ*, tilt angle is* ϕ_k *with angular increment* δ_ϕ*, and z-position is* z_k*.*

A point on lamina k is generated by transforming the corresponding point on the midplane, using the following rotation and translation transformation

$$\begin{bmatrix} x \\ y \\ z \end{bmatrix}_k = \begin{bmatrix} \cos\theta & -\sin\theta\cos\phi & \sin\theta\sin\phi \\ \sin\theta & \cos\theta\cos\phi & -\cos\theta\sin\phi \\ 0 & \sin\phi & \cos\phi \end{bmatrix}_k \begin{bmatrix} x \\ y \\ z \end{bmatrix}_{mid} + \begin{bmatrix} 0 \\ 0 \\ z_k \end{bmatrix} \quad (4)$$

The angular transformation matrix was obtained by multiplying the twist and tilt transformation matrices, $R_z(\theta)R_x(\phi)$, respectively (yaw and roll)[1]. Note that $z_{mid} = 0$ in the above equation.

As observed in the biological tissue, the helicoid is left-handed and its θ ranges from 90° at the bottom lamina to $-90°$ at the top lamina, whereas ϕ ranges from –90° to 90°, respectively, and the angular increments are nearly uniform. Thus, $\Theta = -\Phi = -90°$ in Equation (1), $\delta_\theta = -\delta_\phi = -\pi/(n-1)$ and $\phi_k = -\theta_k$, the same for both twist and tilt but with opposite sense. The lamina thickness t is equivalent to the diameter of the fiber, so that the lamina contains a single layer of fibers. A tightly packed lamina carries $m = w/t$ fibers, and thus m is the number of fibers in each lamina. Numbering the fibers in a lamina from 1 (innermost) to m (outermost), the positions of the endpoints of fiber j in the midplane lamina are $[0 \ \ (j-1)t \ \ 0]^{\mathrm{T}}_{mid}$ and $[l \ \ (j-1)t \ \ 0]^{\mathrm{T}}_{mid}$. Using Equation (4), the z-position of fiber j in lamina k is

$$z_{k,j} = z_k + (j-1)t \sin \phi_k \tag{5}$$

To create the graphical model presented in Figure 5, $\phi_k = -\theta_k$ was substituted in Equation (4), and the transformation was repeated for the fiber edges of all the fibers from 1 to m in all the laminae from 1 to n.

Supplementary Note 2. Spread of fibers in a horizontal cross-section. This note provides the calculation of the angular span of fibers in a horizontal xy cross-section. Refer to the *nesting and interlocking* section in the main text, including Figure 7. xy cross-sections through the BLU (Figure 7d,e) reveal horizontal layers with fan-shaped spreading of fibers, each belonging to a lamina with a different twist angle. The angular span of the fibers in such a virtual layer can be calculated in the following way. Given the layer vertical position z_k, its innermost fiber is twisted θ_k while its outermost fiber belongs to a layer i at position z_i and is twisted θ_i. The distance between these layers is $z_k - z_i \cong w \sin \phi_i$, where $w = mt$ is the lamina width. Expressing z_k in terms of θ_k by combining Equations (1)-(3), $z_k = -\theta_k t(n-1)/\pi$, and substituting $\phi_k = -\theta_k$, we obtain the Equation relating θ_k and θ_i

$$\theta_k \cong \theta_i + \frac{\pi m}{n-1} \sin \theta_i \tag{6}$$

Relating to the example in Figure 7d,e, in the layer $z = 2\mu\text{m}$ (top view) the fibers angle increases from $\theta_k = -68.6°$ to $\theta_i = -39.3°$, whereas in the layer $z = -2\mu\text{m}$ (bottom view) the fibers angle decreases from $\theta_k = 68.6°$ to $\theta_i = 39.3°$. These trends clarify why the fibers spread wider in the upper layer compared to the lower one.

Supplementary Note 3. Approach for laminate analysis. This note provides explanation and justification for the approach taken in the structural analysis. Refer to the *laminate elastic modeling* section in the main text. Classical laminate theory is an obvious choice for such analysis and is a straight forward practical approach, but the question arises whether it is appropriate for such a structure in view of its off-axis, non-parallel and narrow features. To address these concerns, the building scale above the single BLU should be examined. The tight packing of BLUs and the fibrous interlayers and intralayers between BLUs, observed in Figure 2 and modeled in Figure 7, ensure in-plane stress transfer between units so that the laminate can be assumed to be in a plane stress state. Because the interlayers are very thin, their elastic properties do not have a significant effect on the

model and were therefore ignored. Any in-plane cross-section through the Bouligands layer reveals a repeating pattern of fibers (Figure 7d,e), which could be regarded as a sufficiently wide quasi-homogenous lamina. This is also supported by the observation that all the fibers in the laminate remain parallel to the midplane (xy plane) regardless of tilting. Thus, applying classical laminate theory appears justified, with proper adaptations for incorporating the effect of lamina tilting. In the analysis we adopt and briefly describe the method and terms described by Daniel and Ishai[2], where the interested reader can find more details, and highlight the modifications we made. We focus on elastic analysis, and use it to assess the effect of tilt on the laminate stiffness and strength.

Because of the tilt rotation, the corresponding stress at a point in a tilted lamina depends on the point's specific z-position. Consequently, the assumption of laminate theory that the stress is uniform throughout a lamina cannot be applied, and therefore adaptation is required. We considered two approaches: (i) redefining the laminae as horizontal instead of tilted, by taking thin slices parallel to the xy plane in progressive z-positions, as shown in Figure 7d,e; each slice contains a fan-like non-unidirectional arrangement of fibers, with an angular spread of $\theta_k - \theta_i$ (Equation (6)); given the fibers layout, the horizontal lamina is not transversely isotropic, and its stiffness matrix should be recalculated; and (ii) breaking down each tilted lamina to discrete sub-laminae of known average z-coordinates $z_{k,j}$, each having the same stiffness matrix; k denotes a specific lamina within the laminate, and j denotes a specific sub-lamina within lamina k; this approach does not require definition of virtual laminae, and enables calculation within the framework of laminate theory. For simplicity, although not necessary, we can choose the number of sub-laminae in a lamina as m, the number of fibers in a lamina (assumed constant), and therefore $j = 1..m$.

We proceeded with the second approach, and further simplified it by realizing that the laminae in our case are uniform and very thin. This simplification makes it possible to convert the discrete summation of the forces and moments acting on the sub-laminae comprising a single lamina, to continuous integration along the lamina, with negligible deviations.

Supplementary Note 4. Lamina elastic properties. This note provides the calculation of the lamina elastic properties. Refer to the *laminate elastic modeling* section in the main text and to Note 5. The lamina elastic properties are calculated in its own principal material axes $(1,2)$, longitudinal and transversal respectively. Each lamina is a transversely isotropic composite, consisting of tightly-packed unidirectional chitin-protein fibers embedded in a proteinaceous matrix (Figure 2a). The properties of all the laminae comprising the laminate, except for their spatial orientations, are

assumed the same. Given estimated fiber and matrix elastic properties, the lamina elastic properties are calculated by rules of mixtures. As the fibers length in a lamina is bounded by the lamina length and thus not continuous, a shear-lag fiber-length correction factor is applied. The lamina elastic constants lead to a corresponding stiffness matrix $\mathbf{Q}_{1,2}$, which is then transformed to the stiffness matrix $\mathbf{Q}_k = \mathbf{Q}_{x,y}^k$ in the BLU principal axes (x, y) given the lamina twist angle θ_k. Following is the detailed procedure.

The lamina (and fibers) length is l, its thickness is t, the fibers radius is r, and the distance between fiber centers in neighboring laminae is R. Given the fiber and matrix tensile moduli $E_{1\mathrm{f}}$ and E_{m}, the lamina longitudinal modulus is estimated by the rule of mixtures, assuming uniform and equal strain in the longitudinal direction

$$E_1 = \eta_{\mathrm{l}} V_{\mathrm{f}} E_{1\mathrm{f}} + V_{\mathrm{m}} E_{\mathrm{m}} \tag{7}$$

where V_{f} and $V_{\mathrm{m}} = 1 - V_{\mathrm{f}}$ are the fibers and matrix volume fractions, respectively. In a tightly-packed lamina with a single layer of fibers, the volume fraction is the ratio between the fiber cross-sectional area and the square inscribing it, $V_{\mathrm{f}} = \pi/4 = 0.785$. η_{l} is a fiber-length correction factor derived by shear-lag theory[2], which predicts stiffness degradation for short fibers, compared to continuous fibers

$$\eta_{\mathrm{l}} = 1 - \frac{\tanh ns}{ns}, \quad n = \sqrt{\frac{2G_{\mathrm{m}}}{E_{1\mathrm{f}} \ln(R/r)}} \tag{8}$$

where s is the fiber aspect ratio, and G_{m} is the matrix shear modulus. For a tightly-packed lamina with a single layer of fibers, $t = 2r$, $s = l/t$, $R = t$, and $\ln(R/r) = \ln 2$. Assuming uniform and equal stress in the transversal direction, and given the fiber transverse modulus $E_{2\mathrm{f}}$, the lamina transverse modulus is

$$E_2 = \frac{E_{2\mathrm{f}} E_{\mathrm{m}}}{V_{\mathrm{f}} E_{\mathrm{m}} + V_{\mathrm{m}} E_{2\mathrm{f}}} \tag{9}$$

Similarly, assuming constant shear stress, and given the fiber and matrix shear moduli $G_{12\mathrm{f}}$ and G_{m}, the lamina shear modulus is

$$G_{12} = \frac{G_{12\mathrm{f}} G_{\mathrm{m}}}{V_{\mathrm{f}} G_{\mathrm{m}} + V_{\mathrm{m}} G_{12\mathrm{f}}} \tag{10}$$

Finally, given the fiber and matrix Poisson's ratios $\nu_{12\mathrm{f}}$ and ν_{m}, the lamina Poisson's ratios are

$$\nu_{12} = V_f \nu_{12f} + V_m \nu_m \quad \text{and} \quad \nu_{21} = \nu_{12} \frac{E_2}{E_1} \tag{11}$$

The corresponding lamina stiffness matrix is[2]

$$\mathbf{Q}_{1,2} = \begin{bmatrix} E_1/\Delta & \nu_{12}E_2/\Delta & 0 \\ \nu_{12}E_2/\Delta & E_2/\Delta & 0 \\ 0 & 0 & G_{12} \end{bmatrix}, \quad \Delta = \begin{vmatrix} 1 & -\nu_{21} \\ -\nu_{12} & 1 \end{vmatrix} \tag{12}$$

To obtain the lamina stiffness matrix in the BLU principal axes (x, y), the stiffness matrix should be transformed in correspondence to the twist angle θ_k. The transformation matrix for lamina k is $\mathbf{T}_k = \mathbf{T}(\theta_k)$, and as all the laminae are assumed the same, $\mathbf{Q}_{1,2}$ is constant. The transformed stiffness is

$$\mathbf{Q}_k = \mathbf{T}_k^{-1} \mathbf{Q}_{1,2} \mathbf{T}_k^{-\mathrm{T}} \tag{13}$$

where $\mathbf{T}_k^{-\mathrm{T}} = (\mathbf{T}^{\mathrm{T}})^{-1}$ and

$$\mathbf{T}_k = \begin{bmatrix} \cos^2\theta & \sin^2\theta & 2\sin\theta\cos\theta \\ \sin^2\theta & \cos^2\theta & -2\sin\theta\cos\theta \\ -\sin\theta\cos\theta & \sin\theta\cos\theta & \cos^2\theta - \sin^2\theta \end{bmatrix}_k \tag{14}$$

The above transformation accounts only for laminae twisting, and does not account for laminae tilting. However, tilting does not change the spatial orientation of the fibers in a lamina, just their z-position; nor does it change $\mathbf{Q}_k$, as the tilting 1-axis is perpendicular to the lamina plane of isotropy $(2,3)$. Thus, tilting does not affect the transformation. The lamina stiffness matrix $\mathbf{Q}_k$ is independent of the z-position, because all its fibers remain parallel to the midplane after tilting.

The chitin-protein fiber itself is a composite of α-chitin chains embedded in a protein matrix. Thus, the fiber elastic properties, $E_{1f}, E_{2f}, G_{12f}, \nu_{12f}$ are calculated from the α-chitin and protein properties, using the same procedure as described above in Equations (7)-(11).

Supplementary Note 5. Laminate stiffness matrix. This note provides the calculation of the laminate stiffness matrix. Refer to the *laminate elastic modeling* section in the main text and to Notes 3 and 4. The strain at any point in a laminate under load is related to the laminate plane strains $\boldsymbol{\varepsilon}^0 = \left[\varepsilon_x^0 \ \ \varepsilon_y^0 \ \ \gamma_s^0\right]^{\mathrm{T}}$ and curvatures $\boldsymbol{\kappa} = \left[\kappa_x \ \ \kappa_y \ \ \kappa_s\right]^{\mathrm{T}}$ by

$$\boldsymbol{\varepsilon} = \boldsymbol{\varepsilon}^0 + z\boldsymbol{\kappa} \tag{15}$$

where $\boldsymbol{\varepsilon} = [\varepsilon_x \;\; \varepsilon_y \;\; \gamma_s]^{\mathrm{T}}$, two in-plane tensile strains and an in-plane shear strain, and z is the distance from the midplane[2]. The subscript s is a shorthand replacement for xy. Because of the tilt rotation, the corresponding strain and stress at a point in a tilted lamina depends on the point's specific z-position. Consequently, the assumption of laminate theory that the stress is uniform throughout a lamina cannot be applied, and the stress variation within a lamina has to be accounted for. Using the continuous distance s from the z axis as a variable which denotes a position within lamina k (Figure 2a), ranging from 0 to w, the specific z-position at the mid-thickness of lamina k is

$$z_k(s) = z_k + s \sin \phi_k \tag{16}$$

where ϕ_k and z_k are defined in Equations (2) and (3), respectively. The distance s should not be confused with the shear subscript s.

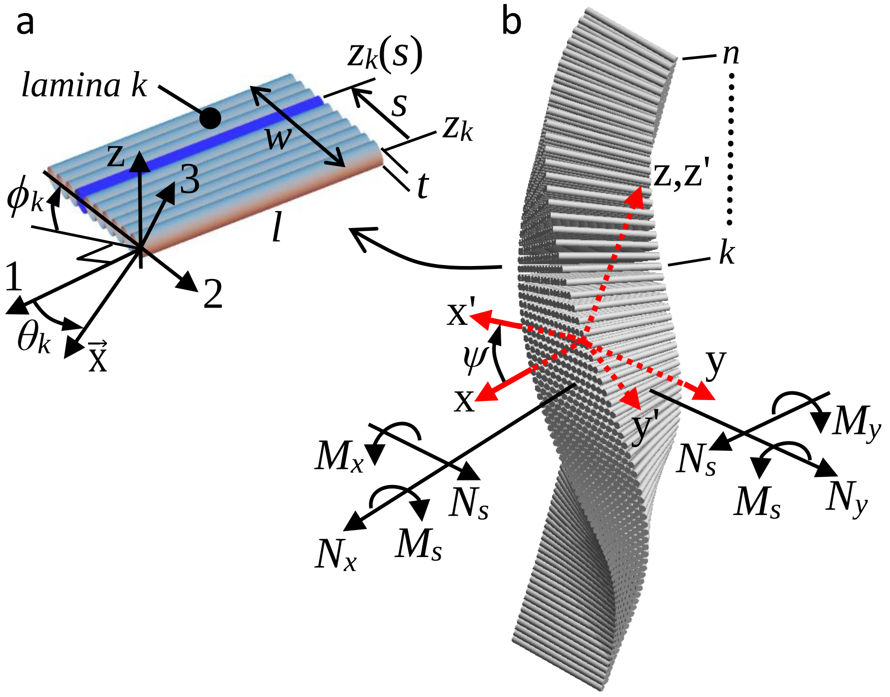

Figure 2. BLU (Bouligand laminate unit) laminate model. *(a) Lamina conformation and rotations. (b) Laminate conformation and overall loads per unit length in the x or y directions: normal forces N_x and N_y, shear force N_s, bending moments M_x and M_y, and torsional moment M_s.*

Given the lamina 3x3 stiffness matrix $\mathbf{Q}_k$ (Equation (13)), the stress at position z in lamina k is (using Equation (15))

$$\boldsymbol{\sigma}_k(s) = \mathbf{Q}_k \boldsymbol{\varepsilon}(s) = \mathbf{Q}_k[\boldsymbol{\varepsilon}^{\mathbf{0}} + z_k(s)\boldsymbol{\kappa}] \tag{17}$$

where $\boldsymbol{\sigma}_k = [\sigma_x \ \ \sigma_y \ \ \tau_s]_k^{\mathrm{T}}$, two in-plane tensile stresses and an in-plane shear stress. $\mathbf{Q}_k$ is obtained from the lamina stiffness matrix in its material axes $(1,2)$, by transforming it to the laminate principal axes (x, y) using the twist angle θ_k. Because the fibers in a lamina are unidirectional, the lamina is transversely quasi-isotropic in its 23 plane, and therefore tilting around the 1-axis does not affect its elastic constants; thus, $\mathbf{Q}_k$ is invariant with respect to the variable s. The detailed calculation of the lamina composite, consisting of tightly-packed unidirectional chitin-protein fibers embedded in a proteinaceous matrix (Figure 2a), is provided in Note 4. The calculation uses rules of mixtures and includes correction for the limited fiber length l.

As the laminae in our case are very thin, we may assume uniform stress across the lamina thickness, and therefore the forces on lamina k per unit length of the laminate (in x or y direction) are $\boldsymbol{\sigma}_k(s)t$. An infinitesimal segment ds contributes a fraction dz/w to the lamina forces, and yields a force fraction $\boldsymbol{\sigma}_k(s)tds/w$. The cumulative loads acting on the laminate (Figure 2b) are calculated by integrating the force fractions on each lamina to obtain their contribution to the laminate forces and moments, and summing up the contribution from all laminae. Thus, the overall forces per unit length of the laminate are given by

$$\mathbf{N} = \frac{t}{w}\sum_{k=1}^{n}\int_0^w \boldsymbol{\sigma}_k(s)ds \tag{18}$$

where $\mathbf{N} = [N_x \ \ N_y \ \ N_s]^{\mathrm{T}}$, two in-plane normal forces and an in-plane shear force. The integration interval is from the inner edge of the lamina to its outer edge (Figure 2a). Similarly, the moment fractions on lamina k are $[\boldsymbol{\sigma}_k(s)tds/w]z_k(s)$, and therefore the overall moments per unit length are

$$\mathbf{M} = \frac{t}{w}\sum_{k=1}^{n}\int_0^w \boldsymbol{\sigma}_k(s)z_k(s)ds \tag{19}$$

where $\mathbf{M} = [M_x \ \ M_y \ \ M_s]^{\mathrm{T}}$, two bending moments and a torsional moment. This calculation leaves small gaps between adjacent laminae as a result of the tilt angle difference between them. These gaps, seen in Figure 2 in the main text, are assumed to be filled by matrix and their contribution to the BLU stiffness is negligible and therefore ignored in the analysis; however, the presence of matrix in these gaps is essential to ensure stress transfer between laminae. Note that, unlike a laminate with parallel laminae, the forces and moments per unit length are functions of the laminate width w.

In case the gaps contribution is desired, the following calculation outline can be used: The matrix in a gap is not reinforced and therefore isotropic. Thus, the stiffness matrix $\mathbf{Q}_k$ in Equation

(17) can be replaced by $\mathbf{Q}_{1,2}$ from Equation (12), which does not vary with the twist angle, where we substitute the matrix properties $E_1 = E_2 = E_\mathrm{m}$, $G_{12} = G_\mathrm{m}$ and $\nu_{12} = \nu_{21} = \nu_\mathrm{m}$. A gap has the shape of a wedge with angle δ_ϕ (Equation (1)), and is the same throughout the laminate. Hence, its local thickness is $s\delta_\phi$, varying with the distance s from the z-axis. This varying thickness can replace the constant thickness t in Equations (18) and (19). Also, the filled gaps slightly degrade the shear-lag length correction factor, η_l, by increasing the ratio R/r in Equation (8) for fibers that are distant from the z-axis. However, after averaging over the whole lamina, this degradation is minor and can be neglected.

Substituting $\boldsymbol{\sigma}_k(s)$ from Equation (17) into the forces and moments equations, we get

$$\mathbf{N} = \frac{t}{w}\sum_{k=1}^{n} \mathbf{Q}_k \int_0^w [\boldsymbol{\varepsilon}^\mathbf{0} + z_k(s)\boldsymbol{\kappa}]ds \tag{20}$$

where $\mathbf{Q}_k$ was taken out of the integral because it is independent of the s-position. As $\boldsymbol{\varepsilon}^\mathbf{0}$ and $\boldsymbol{\kappa}$ are also independent of the s-position, we can rewrite:

$$\mathbf{N} = \frac{t}{w}\sum_{k=1}^{n} \mathbf{Q}_k \left[\boldsymbol{\varepsilon}^\mathbf{0} \int_0^w ds + \boldsymbol{\kappa}\int_0^w z_k(s)ds\right] \tag{21}$$

Similarly,

$$\mathbf{M} = \frac{t}{w}\sum_{k=1}^{n} \mathbf{Q}_k \int_0^w [\boldsymbol{\varepsilon}^\mathbf{0} + z_k(s)\boldsymbol{\kappa}]z_k(s)ds \tag{22}$$

rewritten as

$$\mathbf{M} = \frac{t}{w}\sum_{k=1}^{n} \mathbf{Q}_k \left[\boldsymbol{\varepsilon}^\mathbf{0} \int_0^w z_k(s)ds + \boldsymbol{\kappa}\int_0^w z_k(s)^2 ds\right] \tag{23}$$

Rearranging the forces and moments equations, we get

$$\begin{aligned} \mathbf{N} &= \mathbf{A}\boldsymbol{\varepsilon}^\mathbf{0} + \mathbf{B}\boldsymbol{\kappa} \\ \mathbf{M} &= \mathbf{B}\boldsymbol{\varepsilon}^\mathbf{0} + \mathbf{D}\boldsymbol{\kappa} \end{aligned} \tag{24}$$

where the 3x3 stiffness matrices are

$$\mathbf{A} = \frac{t}{w}\sum_{k=1}^{n} \mathbf{Q}_k \int_0^w ds$$

$$\mathbf{B} = \frac{t}{w}\sum_{k=1}^{n} \mathbf{Q}_k \int_0^w z_k(s) ds \tag{25}$$

$$\mathbf{D} = \frac{t}{w}\sum_{k=1}^{n} \mathbf{Q}_k \int_0^w z_k(s)^2 ds$$

Substituting $z_k(s)$ from Equation (16) , the integrals can be calculated, yielding

$$\mathbf{A} = t\sum_{k=1}^{n} \mathbf{Q}_k$$

$$\mathbf{B} = t\sum_{k=1}^{n} \mathbf{Q}_k \left(z_k + \frac{1}{2} w \sin\phi_k\right) \tag{26}$$

$$\mathbf{D} = t\sum_{k=1}^{n} \mathbf{Q}_k \left(z_k^2 + z_k w \sin\phi_k + \frac{1}{3} w^2 \sin^2\phi_k\right)$$

The terms containing ϕ_k are due to the tilting of laminae, that is they vanish without tilting, and their impact on the matrices grows with $|\phi_k|$, toward the upper and lower regions of the BLU. The solution reduces to the classical form for thin laminae when there is no tilt ($\phi_k = 0$).

The matrix **A** is not affected by tilting, and is in fact the same as in classical laminate theory. The reason for this is that **A** contains in-plane (extension and shear) stiffness components, which are not affected by tilting because tilting of a lamina does not change its fibers twist angle. However, the elastic moduli do change because of the larger overall height H of the BLU due to tilting (see Equation (30). By contrast, **B** and **D** are significantly different because of the integration over a wide range of z within a single lamina. **D** contains flexure (bending and torsion) stiffnesses and **B** contains in-plane/flexure coupling stiffnesses, both affected by the varying values of z. The laminate is of the balanced antisymmetric type, meaning that it consists of pairs of identical θ_k and $-\theta_k$ laminae, arranged at equal z-distance from the midplane. This conformation leads to $A_{xs} = A_{ys} = 0$ (no extension/ shear coupling), $D_{xs} = D_{ys} = 0$ (no bending/torsion coupling), and $B_{xx} = B_{yy} = B_{xy} = B_{ss} = 0$ (no extension/bending coupling), which can be seen in calculated examples. These couplings do occur when the principal axes are rotated as shown in Figure 8 in the main text ($x'y'$ system, rotated by an angle ψ with respect to the xy system).

Supplementary Note 6. Laminate elastic properties. This note provides the calculation of the laminate elastic engineering properties. Refer to the *laminate elastic modeling* section in the main text, including Table 1 and Figure 8, and to Note 5. Extracting these properties directly from the laminate stiffness matrix yields complicated expressions, and therefore using the laminate compliance matrix is preferable[2]. The laminate 6x6 compliance matrix is obtained by inverting the stiffness matrix

$$\begin{bmatrix} \boldsymbol{\varepsilon}^{\mathbf{0}} \\ \boldsymbol{\kappa} \end{bmatrix} = \begin{bmatrix} \mathbf{a} & \mathbf{b} \\ \mathbf{c} & \mathbf{d} \end{bmatrix} \begin{bmatrix} \mathbf{N} \\ \mathbf{M} \end{bmatrix}, \qquad \begin{bmatrix} \mathbf{a} & \mathbf{b} \\ \mathbf{c} & \mathbf{d} \end{bmatrix} = \begin{bmatrix} \mathbf{A} & \mathbf{B} \\ \mathbf{B} & \mathbf{D} \end{bmatrix}^{-1} \tag{27}$$

In the case of in-plane loading (only forces, no moments), the force per unit length is $\mathbf{N} = H\bar{\boldsymbol{\sigma}}$, where $\bar{\boldsymbol{\sigma}} = \begin{bmatrix} \bar{\sigma}_x & \bar{\sigma}_y & \bar{\tau}_s \end{bmatrix}^{\mathrm{T}}$ are the average laminate stresses, and $H = h + 2(w - t)$ is the overall height of the BLU after tilting ($h = nt$ is the net height without tilting). The plane strains reduce to

$$\boldsymbol{\varepsilon}^{\mathbf{0}} = \mathbf{aN} = H\mathbf{a}\bar{\boldsymbol{\sigma}} \tag{28}$$

The effective engineering constants of the laminate can be obtained by separately calculating the strains for each of the loading conditions $\bar{\sigma}_x$, $\bar{\sigma}_y$ and $\bar{\tau}_s$, and then superposing them, yielding[2]

$$\mathbf{a} = \frac{1}{H} \begin{bmatrix} \frac{1}{\bar{E}_x} & -\frac{\bar{\nu}_{yx}}{\bar{E}_y} & \frac{\bar{\eta}_{sx}}{\bar{G}_{xy}} \\ -\frac{\bar{\nu}_{xy}}{\bar{E}_x} & \frac{1}{\bar{E}_y} & \frac{\bar{\eta}_{sy}}{\bar{G}_{xy}} \\ \frac{\bar{\eta}_{xs}}{\bar{E}_x} & \frac{\bar{\eta}_{ys}}{\bar{E}_x} & \frac{1}{\bar{G}_{xy}} \end{bmatrix} \tag{29}$$

Given $\mathbf{a}$ calculated by Equation (27), the corresponding terms in both stiffness matrices are equated, and the laminate effective engineering constants are derived. The Young's moduli in the x and y directions and the shear modulus are

$$\bar{E}_x = \frac{1}{Ha_{xx}} \qquad \bar{E}_y = \frac{1}{Ha_{yy}} \qquad \bar{G}_{xy} = \frac{1}{Ha_{ss}} \tag{30}$$

the Poisson's ratios relating normal strains to orthogonal normal stresses are

$$\bar{\nu}_{xy} = -\frac{\varepsilon_y^0}{\varepsilon_x^0} = -\frac{a_{yx}}{a_{xx}} \qquad \bar{\nu}_{yx} = -\frac{\varepsilon_x^0}{\varepsilon_y^0} = -\frac{a_{xy}}{a_{yy}} \tag{31}$$

the shear coupling coefficients relating shear strains to normal stresses are

$$\bar{\eta}_{xs} = \frac{\gamma_s^0}{\varepsilon_x^0} = \frac{a_{sx}}{a_{xx}} \qquad \bar{\eta}_{ys} = \frac{\gamma_s^0}{\varepsilon_y^0} = \frac{a_{sy}}{a_{yy}} \tag{32}$$

and the shear coupling coefficients relating normal strains to shear stresses are

$$\bar{\eta}_{sx} = \frac{\varepsilon_x^0}{\gamma_s^0} = \frac{a_{xs}}{a_{ss}} \qquad \bar{\eta}_{sy} = \frac{\varepsilon_y^0}{\gamma_s^0} = \frac{a_{ys}}{a_{ss}} \tag{33}$$

Similarly, in the case of flexural loading (only moments, no forces), the curvatures in Equation (27) reduce to

$$\boldsymbol{\kappa} = \mathbf{dM} \tag{34}$$

Defining the flexural stiffness as $K = wM/\kappa$, where wM is the overall moment acting on the laminate width w, we get the effective bending stiffnesses

$$\bar{K}_x = \frac{w}{d_{xx}} \qquad \bar{K}_y = \frac{w}{d_{yy}} \qquad \bar{K}_s = \frac{w}{d_{ss}} \tag{35}$$

There are flexural cross couplings as well, which are not shown here.

Supplementary Note 7. Effect of tilt on laminate strength. This note provides the calculation of the effect of tilt on the laminate strength. Refer to the *laminate strength and toughness* section in the main text and to Note 6. The laminate strength can be assessed by considering the conservative first ply failure (FPF) criterion[2]. Substituting $\boldsymbol{\varepsilon}^0$ and $\boldsymbol{\kappa}$ from Equation (27) in Equation (15), we obtain the strains at any z-position in the laminate

$$\boldsymbol{\varepsilon} = [\mathbf{a} + z\mathbf{c}]\mathbf{N} + [\mathbf{b} + z\mathbf{d}]\mathbf{M} \tag{36}$$

The factor $\mathbf{a} + z\mathbf{c}$ determines the strains under in-plane loads, whereas $\mathbf{b} + z\mathbf{d}$ determines the strains under flexural loads. To obtain the stresses in a lamina, the strains should be referred to the lamina principal material axes $(1,2)$, using the transformation $\mathbf{T}_k$ in Equation (14), taking into account the specific z-position and twist angle of each lamina. To determine which is the critical lamina in terms of strength, this calculation should be repeated on all laminae. However, this is not necessary for evaluating the effect of tilting on the strength, as demonstrated in the examples in the main text.

Similar analysis can be carried out to determine the effect of tilting on the interlaminar shear strength under a shear load in the z-direction. As a result of such force, the flexural moments $\mathbf{M}$ in

Equation (36) vary with respect to the position in the laminate, and consequently the laminate strains will vary compatibly. These in-plane stress gradients, which are proportional to the factor $\mathbf{b} + z\mathbf{d}$, are balanced by interlaminar shear stresses, and therefore the effect of tilting on the interlaminar strength under such loading condition should be similar to that under flexural loads.

To determine whether failure would occur under a combination of stresses, a failure criterion should be applied. For composites reinforced by strong and stiff fibers, such as the BLU, the Hashin-Rotem criterion can be applied, which separates the fiber and interfiber failure modes in a lamina:[2]

$$\frac{|\sigma_1|}{F_1} = 1$$
$$\left(\frac{\sigma_2}{F_2}\right)^2 + \left(\frac{\tau_6}{F_6}\right)^2 = 1 \qquad (37)$$

where $\sigma_1, \sigma_2, \tau_6$ are the principal stresses in the lamina, and F_1, F_2, F_6 are its corresponding strengths. In the case of the middle lamina and the top and bottom laminae, the laminate principal axes coincide with those of the lamina. This allows laminate strength assessment without the need for angular transformation of the strains. For example, in these specific laminae, an increased shear stress combined with an unchanged normal stress would result in decreasing the strength. Conversely, a decreased shear stress combined with an unchanged or decreased normal stress would result in increasing the strength. Both examples are demonstrated in the main text.

Supplementary Note 8. Fracture type of the cuticle samples. This note expands on the fracture type of the cuticle samples. Refer to the *BLU structures in the tarsus cuticle* section in the main text including Figure 2. The preparation of the SEM samples was done by manually breaking them following a well-defined and proven protocol (see Methods), rather than cutting them and inducing unwanted deformations. Images were taken from 3 different adult animals, all showing the same typical structure. The question that arises is whether the BLU conformation seen in the SEM images in Figure 2 is the result of a crack propagating internally through the BLU due to residual stresses, or the result of a separation between adjacent BLUs. The answer to this question becomes clearer when observing the view in Figure 3. We see a repeatable pattern of three layers, each with a sequence of nested BLUs. The BLUs seem intact, without debris that might have indicated internal fractures, and without the erratic paths that are typical of crack propagation. The BLU laminae are seen twisted and tilted, repeatedly in each BLU with the same pattern. There are no signs indicating that either twisting or tilting might have been caused by deformation induced during the breaking of

the cuticle. Furthermore, if the BLUs were internally fractured it would mean that they are substantially thicker than viewed, but when observing the longitudinal cross section in Figure 2d, the BLUs are seen to be very slim to the extent that an internal crack propagating along their height is improbable. Put together with the images in Figure 2, we may conclude that the breaking of the cuticle causes separation between BLUs at the intralayer rather than fracture of the BLU itself.

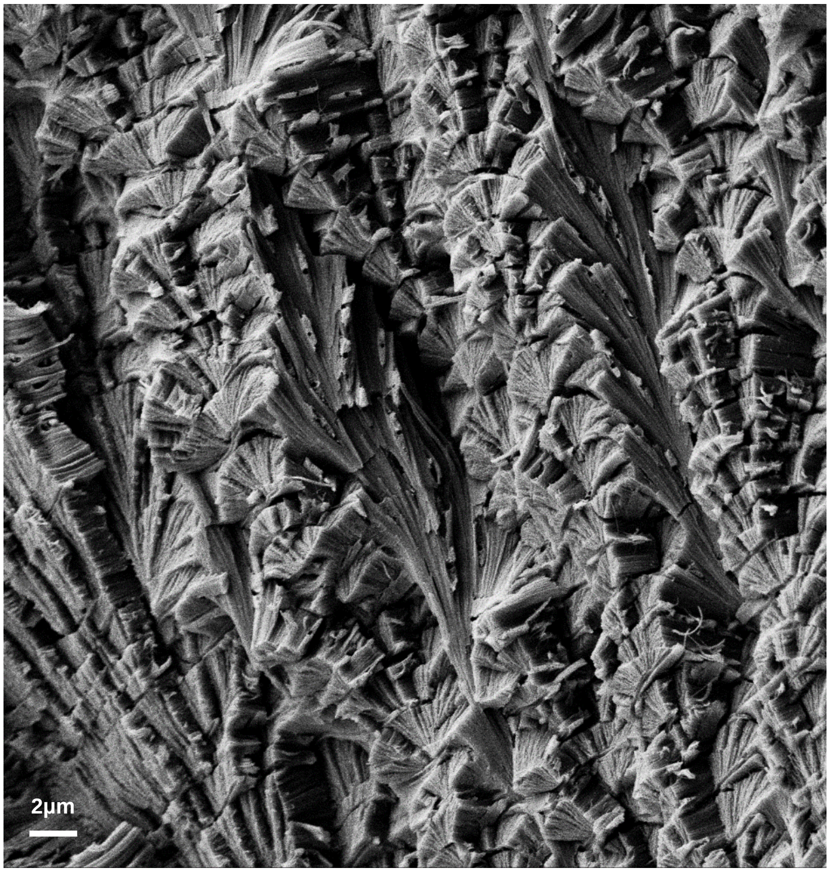

Figure 3. Oblique SEM view of layers of nested BLUs (Bouligand laminate units). *Zoom-out of the region shown in Figure 2f in the main text.*

References

1 Weisstein, E. W. *Euler Angles, From MathWorld-A Wolfram Web Resource,* http://mathworld.wolfram.com/EulerAngles.html

2 Daniel, I. M. & Ishai, O. *Engineering Mechanics of Composite materials*. (Oxford, 2006).
