# Supplementary material for: Nested helicoids in biological microstructures
Source: Nat Commun. 2020 Jan 13;11:224. doi: 10.1038/s41467-019-13978-6 (PMC6957508; doi:10.1038/s41467-019-13978-6)
Supplement: Supplementary file 3 — Description of Additional Supplementary Files [file 41467_2019_13978_MOESM3_ESM.pdf]

Description of Additional Supplementary Files

Supplementary Movie 1: “Nested helicoids in biological microstructures – graphical animation describing the laminating, off-axis twisting, and tilting comprising the Bouligand unit, and the nesting and layering of multiple units.”
