# Supplementary material for: Nested helicoids in biological microstructures
Source: Nat Commun. 2020 Jan 13;11:224. doi: 10.1038/s41467-019-13978-6 (PMC6957508; doi:10.1038/s41467-019-13978-6)
Supplement: Supplementary file 5 — Reporting Summary [file 41467_2019_13978_MOESM5_ESM.pdf]

nature research

Corresponding author(s): Israel Greenfeld

Last updated by author(s): Nov 25, 2019

Reporting Summary

Nature Research wishes to improve the reproducibility of the work that we publish. This form provides structure for consistency and transparency in reporting. For further information on Nature Research policies, see Authors & Referees and the Editorial Policy Checklist .

Statistics

For all statistical analyses, confirm that the following items are present in the figure legend, table legend, main text, or Methods section.

n/a	Confirmed	
☒	☐	The exact sample size (*n*) for each experimental group/condition, given as a discrete number and unit of measurement
☒	☐	A statement on whether measurements were taken from distinct samples or whether the same sample was measured repeatedly
☒	☐	The statistical test(s) used AND whether they are one- or two-sided<br>*Only common tests should be described solely by name; describe more complex techniques in the Methods section.*
☒	☐	A description of all covariates tested
☒	☐	A description of any assumptions or corrections, such as tests of normality and adjustment for multiple comparisons
☒	☐	A full description of the statistical parameters including central tendency (e.g. means) or other basic estimates (e.g. regression coefficient) AND variation (e.g. standard deviation) or associated estimates of uncertainty (e.g. confidence intervals)
☒	☐	For null hypothesis testing, the test statistic (e.g. *F*, *t*, *r*) with confidence intervals, effect sizes, degrees of freedom and *P* value noted<br>*Give P values as exact values whenever suitable.*
☒	☐	For Bayesian analysis, information on the choice of priors and Markov chain Monte Carlo settings
☒	☐	For hierarchical and complex designs, identification of the appropriate level for tests and full reporting of outcomes
☒	☐	Estimates of effect sizes (e.g. Cohen's *d*, Pearson's *r*), indicating how they were calculated

Our web collection on statistics for biologists contains articles on many of the points above.

Software and code

Policy information about availability of computer code

Data collection	SEM and TEM images were taken from scorpion cuticle samples
Data analysis	A custom Mathcad program was used for the laminate model calculations. No software was used for data collection or analysis. Statement was added in the manuscript: "Code availability: All relevant codes are available from the authors."

For manuscripts utilizing custom algorithms or software that are central to the research but not yet described in published literature, software must be made available to editors/reviewers. We strongly encourage code deposition in a community repository (e.g. GitHub). See the Nature Research guidelines for submitting code & software for further information.

Data

Policy information about availability of data

All manuscripts must include a data availability statement. This statement should provide the following information, where applicable:

- Accession codes, unique identifiers, or web links for publicly available datasets
- A list of figures that have associated raw data
- A description of any restrictions on data availability

Data availability: All relevant data are available from the authors.

Field-specific reporting

Please select the one below that is the best fit for your research. If you are not sure, read the appropriate sections before making your selection.

☐ Life sciences ☐ Behavioural & social sciences ☒ Ecological, evolutionary & environmental sciences

Ecological, evolutionary & environmental sciences study design

All studies must disclose on these points even when the disclosure is negative.

Study description	Geometrical analysis of the helical laminated structure (named Bouligand) of the scorpion tarsus cuticle (moveable claw), and modeling of its mechanical properties. The goal is to characterize the properties of these complex biological nanostructures, as inspiration for future synthetic composite structures.
Research sample	The samples were taken from the species Scorpio Maurus Palmatus. The cuticle of this species include the Bouligand helicoidal laminate structures, which are the subject of the study.
Sampling strategy	SEM and TEM images were obtained from 3 different animals, yielding the same typical helicoidal nanostructure.
Data collection	Adult scorpions belonging to the species Scorpio Maurus Palmatus were collected from the area of Sde Boker in the Negev desert in the south of Israel, in collaboration with the Israeli Parks and Nature Authority and the Hoopoe Yeruham Ornithology and Ecology Center. The scorpions were transported separately in plastic flasks that were sealed inside a hard-plastic box, to prevent escape during conveyance. The scorpions were submerged in liquid nitrogen and then stored in plastic boxes in a freezer at -80 °C. Scorpion chelae were mechanically separated from the rest of the body before characterization. The data was collected by Israel Kellersztein, a co-author of the manuscript.
Timing and spatial scale	The scorpions were collected within a short span of time in the same field region.
Data exclusions	No data were excluded from the analysis.
Reproducibility	The procedure is reproducible, as described in detail in the Methods section in the manuscript.
Randomization	Not relevant for the structural analysis performed in this study.
Blinding	Not relevant for the structural analysis performed in this study.

Did the study involve field work? ☐ Yes ☒ No

Reporting for specific materials, systems and methods

We require information from authors about some types of materials, experimental systems and methods used in many studies. Here, indicate whether each material, system or method listed is relevant to your study. If you are not sure if a list item applies to your research, read the appropriate section before selecting a response.

Materials & experimental systems

n/a	Involved in the study	
☒	☐	Antibodies
☒	☐	Eukaryotic cell lines
☒	☐	Palaeontology
☐	☒	Animals and other organisms
☒	☐	Human research participants
☒	☐	Clinical data

Methods

n/a	Involved in the study	
☒	☐	ChIP-seq
☒	☐	Flow cytometry
☒	☐	MRI-based neuroimaging

Animals and other organisms

Policy information about studies involving animals; ARRIVE guidelines recommended for reporting animal research

Laboratory animals	The study did not involve live laboratory animals.
Wild animals	Adult scorpions belonging to the species Scorpio Maurus Palmatus were collected from the area of Sde Boker in the Negev desert in the south of Israel, in collaboration with the Israeli Parks and Nature Authority. The scorpions were transported separately in plastic flasks that were sealed inside a hard-plastic box, to prevent escape during conveyance. The scorpions were submerged in liquid nitrogen and then stored in plastic boxes in a freezer at -80 °C.
Field-collected samples	The study did not involve samples collected in the field.
Ethics oversight	The Israeli law "Animal suffering (experiments on animals), 1994" (https://www.health.gov.il/LegislationLibrary/Veter02.pdf), excludes invertebrate animals (including scorpions), and therefore no formal ethical approval was required to conduct the experiments. The law is available in Hebrew only, and in the Definitions section in the Introduction the relevant definition is: "In this law – 'Animal' is a vertebrate except human". There is no supplement to this law nor a separate law regarding experiments

on invertebrates.

Note that full information on the approval of the study protocol must also be provided in the manuscript.
